# Microlearning for surgical residents enhances perioperative comprehensive geriatric assessment

**DOI:** 10.1111/jgs.18612

**Published:** 2023-09-29

**Authors:** Janani Thillainadesan, Vasi Naganathan, Sarah N. Hilmer, Richard Kerdic, Sarah J. Aitken

**Affiliations:** ^1^ Department of Geriatric Medicine Concord Hospital Concord, Sydney New South Wales Australia; ^2^ Faculty of Medicine and Health The University of Sydney Camperdown, Sydney New South Wales Australia; ^3^ Centre for Education and Research on Ageing Concord Hospital Sydney New South Wales Australia; ^4^ Kolling Institute of Medical Research The University of Sydney and Northern Sydney Local Health District St Leonards, Sydney New South Wales Australia; ^5^ Department of Vascular Surgery Concord Hospital Concord, Sydney New South Wales Australia; ^6^ Concord Institute of Academic Surgery Concord Hospital Concord, Sydney New South Wales Australia

**Keywords:** comprehensive geriatric assessment, delirium, education, frailty, perioperative care

## BACKGROUND

Geriatrician‐led implementation of comprehensive geriatric assessment and management (CGA) of older surgical patients (e.g., in comanagement models) improves patient care and outcomes.^1^ However, there are no enough geriatricians to meet the demand for comanagement services. There is a need to upskill the workforce in core geriatric competencies, including residents looking after older surgical patients.^2^

Residents have a central role in implementing care processes for older inpatients. Tailoring education to their different learning skills and behaviors provides an opportunity to improve outcomes of older surgical patients. Millennial and Gen Z residents are multitaskers and technology savvy, have shorter learning attention spans, and prefer active learning modes such as observational and experiential learning.

Microlearning, an emerging strategy for training health professionals, targets single learning objectives and is conveniently accessed through digital platforms at the learner's preferred time and place.^3^ While it has demonstrated benefits when compared to traditional teaching methods, microlearning has not been evaluated in geriatric education for residents.

We introduced a novel model of care, Geriatric Comanagement of Older Vascular Surgery Patients (GeriCO‐V), which embedded a geriatrician within the vascular surgery team, who had responsibilities for patient care and resident education. We evaluated the impact of the microlearning educational intervention on residents' performance of key care processes.

## METHODS

The study was conducted at a tertiary hospital with a busy vascular surgery unit. The study received ethics approval from the local Human Research Ethics Committee.

Our educational intervention for residents on the vascular surgery team was a novel aspect of the geriatric comanagement model of care. Prior to the introduction of geriatric comanagement, no formal teaching was provided on geriatric principles and practices for these residents. These residents are in postgraduate years (PGYs) 1–3 (mostly PGY1) and are allocated a three‐month rotation in vascular surgery as part of their mandatory broad‐based training years (similar to the transitional year program in North America).

For the educational intervention, the vascular surgery and geriatric medicine departments identified the assessment and management of frailty and delirium as key areas for improvement. Screening for frailty and delirium is recommended by international perioperative care guidelines (e.g., American College of Surgeons and American Geriatrics Society) yet is infrequently performed.^1^

A blended teaching model using targeted microlearning with ward‐based experiential methods was implemented by the geriatrician. Microlearning involved the teaching of two microunits of knowledge and skills: (1) screening for delirium and cognitive impairment using the 4 ‘A's Test (4AT) and (2) screening for frailty using the Clinical Frailty Scale (CFS).

The geriatrician provided a 15‐min teaching session on cognitive impairment, and delirium and frailty screening using the 4AT and CFS instruments. Residents received 4AT and CFS lanyard cards and were directed to mobile application (apps) versions (Supplementary Figure S1). Mobile apps were chosen as they are freely available on the go, their use can be modeled on ward rounds, and these apps allow for real time entry of patient data to determine delirium and frailty scores. Other CGA components such as deprescribing and advance care planning were taught on‐the‐go during ward rounds.

The impact of the educational intervention was assessed by measuring the occurrence of 12 care processes for which the residents were responsible (Supplementary Table S1). Trained independent chart abstractors collected these data. The pre‐ and postintervention frequencies of care processes were compared using Pearson chi‐squared tests and Fisher's exact test.

## RESULTS

Charts from 302 vascular surgery patients aged 65 years or older (preintervention 150 and postintervention 152) were audited for care processes performed by the residents (Supplementary Table S2).

During the preintervention period, there were in total eight vascular surgery residents: four female, seven PGY1, and one PGY3. During the postintervention period, there were 12 residents: five female, eight PGY1, and four PGY2/3.

A significant increase was observed in the occurrence of 9 of the 12 care processes during the postintervention period (Table 1). The largest improvements were seen in rates of screening for frailty (0.0% vs. 64.5%), cognitive impairment (8.0% vs. 76.3%) and delirium (2.0% vs. 69.1%), and documentation of mobility status at admission (26.7% vs. 78.3%).

**TABLE 1:** Occurrence of care processes performed by residents before and after implementation of targeted geriatrics education.

Care process	Proportion of patients^a^ (%)		*p*‐value
	Preintervention (*n* = 150)	Postintervention (*n* = 152)	
Documentation‐related			
Documented pain status at admission	92/150 (61.3)	105/152 (69.1)	0.158
Documented medications at admission	80/150 (53.3)	113/152 (74.3)	<0.001
Documented alcohol and/or smoking history at admission	61/150 (40.7)	105/152 (69.1)	<0.001
Documented functional status at admission	51/150 (34.0)	115/152 (75.7)	<0.001
Documented mobility status at admission	40/150 (26.7)	119/152 (78.3)	<0.001
Documented cause for delirium where the doctor has documented a delirium	8/10 (80.0)	12/14 (85.7)	0.711
Documented medical review post‐fall in hospital including prodromal symptoms and medication review	3/6 (50.0)	4/7 (57.1)	0.797
Screening and/or assessment related			
Screening for frailty using the Clinical Frailty Scale	0/150 (0.0)	98/152 (64.5)	<0.001
Screening for cognitive impairment in hospital	12/150 (8.0)	116/152 (76.3)	<0.001
Screening for delirium in hospital	3/150 (2.0)	105/152 (69.1)	<0.001
Cognition screen at preadmission outpatient clinic	7/47 (14.9)	17/30 (56.7)	<0.001
Delirium screening at preadmission outpatient clinic	10/47 (21.3)	15/30 (50.0)	0.009

^a^ Care processes were only assessed in patients in whom the care process was indicated.

## DISCUSSION

Implementation of care processes based on geriatric medicine principles is recommended to reduce medical and geriatric syndrome complications in the older surgical population.^1^ Yet many of these processes are not performed routinely. Here, we successfully addressed this challenge by implementing targeted geriatrics education for residents. Innovative training methods are required to improve competence in the care of older adults, and not just for those training in geriatrics but across residency programs.

Microlearning combined with ward‐based experiential learning effectively improved residents' clinical practice. We observed dramatic increases in completed care processes performed by residents. Screening for frailty, previously not performed at all, rose to 65%, and prevalence of screening for cognitive impairment and delirium increased from 8% to 76%, and 2% to 69%, respectively.

Microlearning, aligning with Millennial and Gen Z preferences, delivers short and focused teaching through accessible formats.^3^ It can be combined with experiential learning during ward rounds, effectively enhancing multiple competencies through participation in a single patient care activity.^4^ This study demonstrated the effectiveness of microlearning in geriatric education for busy surgical residents, indicating its potential application in other clinical settings. Microlectures and freely available mobile applications provide rapid access to educational content and facilitate timely clinical assessments. The mobile application is a self‐contained reusable learning object that is accessible to learners anywhere and anytime.^3^

A key limitation of this study is that as a blended educational intervention, we cannot establish whether microlearning, experiential learning, or both were necessary for the impact observed. Qualitative methods of investigation are required to explore whether and why there were changes at the clinician behavior level, and longitudinal follow‐up of residents' knowledge and behaviors to determine retention of learning.

## CONCLUSIONS

Key components of CGA can be taught to residents using microlearning and ward‐based experiential learning methods. Geriatric research needs to prioritize how to implement and evaluate education for the wider workforce. This involves harnessing innovative pedagogy aligned with the learning context of today's health professional learners.

## AUTHOR CONTRIBUTIONS

All authors participated in study concept and design, data analysis and interpretation, and the preparation of the manuscript. Thillainadesan managed the acquisition of data.

## FUNDING INFORMATION

This work was supported by Sydney Local Health District's, The Pitch Project, and Ageing and Alzheimer's Institute. Dr Thillainadesan was supported by the National Health and Medical Research Council (NHMRC) Medical Research Postgraduate Scholarship, and MIGA's Doctors in Training Grants Program.

## CONFLICT OF INTEREST STATEMENT

None.

## SPONSOR'S ROLE

None.

## Supporting information


**Supplementary Figure S1.** Photos of lanyards and screenshots of mobile apps.
**Supplementary Table S1.** Measured care processes.
**Supplementary Table S2.** Patient characteristics.
