# Supplementary material for: Microlearning for surgical residents enhances perioperative comprehensive geriatric assessment
Source: J Am Geriatr Soc. 2023 Sep 29;71(12):E30–3. doi: 10.1111/jgs.18612 (PMC10952399; doi:10.1111/jgs.18612)
Supplement: Supplementary file 1 — Supplementary Figure S1. Photos of lanyards and screenshots of mobile apps. Supplementary Table S1. Measured care processes. Supplementary Table S2. Patient characteristics. [file JGS-71-E30-s001.pdf]

Supplementary Figure S1. Photos of lanyards and screenshots of mobile apps

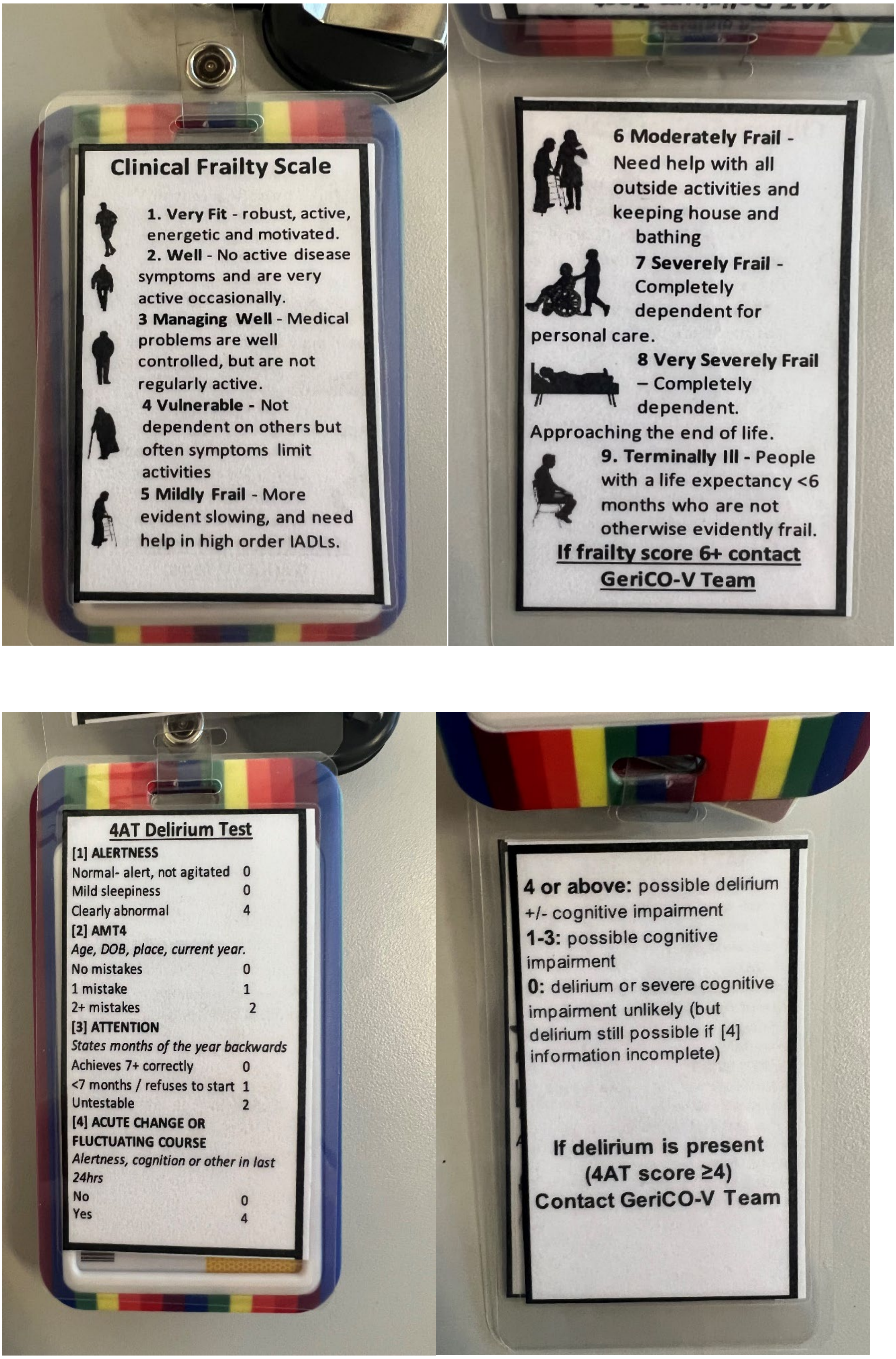

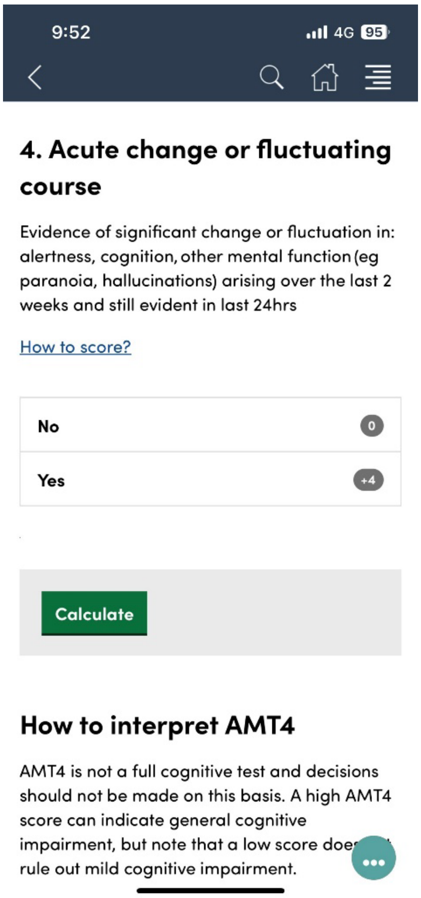
9:52
4G 95
4. Acute change or fluctuating course
Evidence of significant change or fluctuation in: alertness, cognition, other mental function (eg paranoia, hallucinations) arising over the last 2 weeks and still evident in last 24hrs
How to score?
No 0
Yes +4
Calculate
How to interpret AMT4
AMT4 is not a full cognitive test and decisions should not be made on this basis. A high AMT4 score can indicate general cognitive impairment, but note that a low score does
rule out mild cognitive impairment.

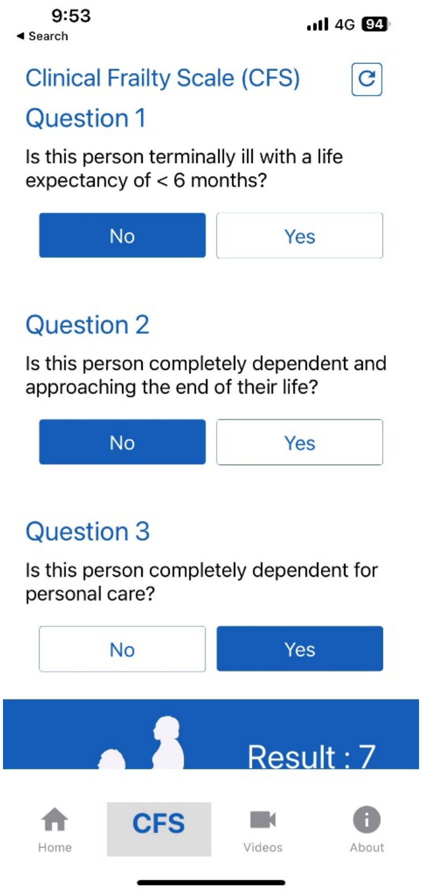
9:53
◂ Search
4G 94
Clinical Frailty Scale (CFS)
Question 1
Is this person terminally ill with a life expectancy of < 6 months?
No
Yes
Question 2
Is this person completely dependent and approaching the end of their life?
No
Yes
Question 3
Is this person completely dependent for personal care?
No
Yes
Result : 7
Home
CFS
Videos
About

Supplementary Table S1. Measured care processes

**Care process performed by residents**
**Documentation-related**
Documented pain status at admission
Documented medications at admission
Documented alcohol and/or smoking history at admission
Documented functional status at admission
Documented mobility status at admission
Documented cause for delirium where the doctor has documented a delirium
Documented medical review post fall in hospital including prodromal symptoms and medication review
**Screening and/or assessment related**
Screening for frailty using the Clinical Frailty Scale
Screening for cognitive impairment in hospital
Screening for delirium in hospital
Cognition screen at pre-admission outpatient clinic
Delirium screening at pre-admission outpatient clinic/vascular nurse specialist

Supplementary Table S2. Patient characteristics

**Characteristic**	**Preintervention (n=150)**	**Postintervention (n=152)**
Age, median (IQR)	80.0 (74.0-84.0)	78.5 (73.0-84.0)
Male, No. (%)	102 (68.0)	100 (65.8)
CALD, No. (%)	47 (31.3)	43 (28.3)
Residential aged care resident, No. (%)	16 (10.7)	25 (16.4)
Current or ex-smoker*, No. (%)	84 (58.3)	87 (58.8)
Presence of frailty (CFS > 4), No. (%)	45 (30.0)	66 (43.4)
Functional dependence (in ≥ 1 ADL), No. (%)	48 (32.0)	58 (38.2)
Cognitive impairment (dementia diagnosis or AMTS score <8), No. (%)	54 (38.6)	48 (33.6)
Assisted mobility, No. (%)	19 (12.7)	24 (15.8)
History of falls, No. (%)	56 (37.3)	71 (46.7)
Underwent operative management, No. (%)	117 (78.0)	100 (65.8)
ASA ≥3, No. (%)	110 (94.0)	97 (97.0)
Charlson score, median (IQR)	3 (2.0-5.0)	3 (2.0-4.3)
Emergency admission, No. (%)	56 (37.3)	72 (47.4)
LOS under vascular surgery inpatient service, geometric mean (95% CI), (days)	6.5 (5.7-7.5)	6.8 (5.9-7.8)
Death during admission, No. (%)	3 (2.0)	2 (1.3)

AMTS, Abbreviated Mental Test Score; ADL, activities of daily living; ASA, American Society of Anesthesiologists physical status classification; CFS, Clinical Frailty Scale; CALD, culturally and linguistically diverse; CI, confidence interval; IQR, interquartile range; LOS, length of stay.

*Data missing (n=10).
